# A Novel Tri-Functionality pH-Magnetic-Photocatalytic Hybrid Organic-Inorganic Polyoxometalates Augmented Microspheres for Polluted Water Treatment

**DOI:** 10.3390/membranes13020174

**Published:** 2023-01-31

**Authors:** Li Ying Yee, Qi Hwa Ng, Siti Kartini Enche Ab Rahim, Peng Yong Hoo, Pei Thing Chang, Abdul Latif Ahmad, Siew Chun Low, Siew Hoong Shuit

**Affiliations:** 1 Faculty of Chemical Engineering & Technology, Universiti Malaysia Perlis (UniMAP), Arau 02600, Perlis, Malaysia; 2 Centre of Excellence for Frontier Materials Research (CFMR), Universiti Malaysia Perlis (UniMAP), Arau 02600, Perlis, Malaysia; 3 School of Chemical Engineering, Engineering Campus, Universiti Sains Malaysia, Seri Ampangan, Nibong Tebal 14300, Pulau Pinang, Malaysia; 4 Department of Chemical Engineering, Lee Kong Chian Faculty of Engineering & Science, Sungai Long Campus, Universiti Tunku Abdul Rahman, Jalan Sungai Long, Bandar Sungai Long, Cheras, Kajang 43000, Selangor, Malaysia; 5 Centre of Photonics and Advanced Materials Research, Sungai Long Campus, Universiti Tunku Abdul Rahman, Jalan Sungai Long, Bandar Sungai Long, Cheras, Kajang 43000, Selangor, Malaysia

**Keywords:** hybrid polyoxometalates, magnetic responsive, photocatalytic responsive, pH-responsive, augmented microspheres

## Abstract

The severe water pollution from effluent dyes threatens human health. This study created pH-magnetic-photocatalytic polymer microspheres to conveniently separate the photocatalyst nanoparticles from the treated water by applying an external magnetic field. While fabricating magnetic nanoparticles’ (MNPs) microspheres, incorporating 0.5 wt.% iron oxide (Fe_3_O_4_) showed the best magnetophoretic separation ability, as all the MNPs microspheres were attracted toward the external magnet. Subsequently, hybrid organic–inorganic polyoxometalates (HPOM), a self-synthesized photocatalyst, were linked with the functionalized magnetic nanoparticles (f-MNPs) to prepare augmented magnetic-photocatalytic microspheres. The photodegradation dye removal efficiency of the augmented magnetic-photocatalytic microspheres (f-MNPs-HPOM) was then compared with that of the commercial titanium dioxide (TiO_2_) photocatalyst (f-MNPs-TiO_2_). Results showed that f-MNPs-HPOM microspheres with 74 ± 0.7% photocatalytic removal efficiency better degraded methylene orange (MO) than f-MNPs-TiO_2_ (70 ± 0.8%) at an unadjusted pH under UV-light irradiation for 90 min. The excellent performance was mainly attributed to the lower band-gap energy of HPOM (2.65 eV), which required lower energy to be photoactivated under UV light. The f-MNPs-HPOM microspheres demonstrated excellent reusability and stability in the photo-decolorization of MO, as the microspheres retained nearly the same removal percentage throughout the three continuous cycles. The degradation rate was also found to follow the pseudo-first-order kinetics. Furthermore, f-MNPs-HPOM microspheres were pH-responsive in the photodegradation of MO and methylene blue (MB) at pH 3 (acidic) and pH 9 (alkaline). Overall, it was demonstrated that using HPOM photocatalysts in the preparation of magnetic-photocatalytic microspheres resulted in better dye degradation than TiO_2_ photocatalysts.

## 1. Introduction

Humanity cannot survive without clean water, whether it be used for drinking, food production, recreation, or household usage [1]. The number of people losing access to essential potable water is predicted to grow as time goes on and as the uncontrolled and unregulated discharge of untreated wastewater containing microbes, organic, and inorganic contaminants from agricultural, industrial, municipal, and domestic wastes enter the water bodies [2]. In particular, dye effluents pose a significant challenge to environmental management due to their high color intensity, fluctuating pH, malodor, high biological oxygen demand (BOD) and chemical oxygen demand (COD), acids and alkali content, as well as containing various heavy metals that violate environmental standards [3,4]. The presence of dye pollutants such as methyl orange (MO) and methylene blue (MB) could result in adverse health effects, including but not limited to skin irritation, nausea, and vomiting in humans, as well as restricting the passage of sunlight for photosynthesis, which is the basis of life on Earth [3,5]. In addition, some of these dyes contain azo-type compounds, which are highly toxic, mutagenic, and potentially carcinogenic [6,7].

Advanced oxidation processes (AOPs) are essential and appealing for degrading pollutants from contaminated water, as they can produce strongly oxidizing radicals under UV light; furthermore, these are mainly hydroxyl radicals (∙OH), which, in turn, can degrade the complex compounds into simple and harmless substances such as CO_2_, H_2_O, and other mineral substances [8,9]. Of the AOPs, TiO_2_, a semiconductor photocatalyst, is frequently used to remove contaminants from the aqueous phase in heterogeneous processes [9]. TiO_2_ nanoparticles offer intriguing properties, such as a high specific surface area-to-volume ratio, strong oxidizing power, and low toxicity [10,11,12].

Even though TiO_2_ nanoparticles have numerous intrinsic features, they suffer from a wide energy band-gap, which is typically around 3.2 eV in the anatase phase and 3.0 eV in the rutile phase [11,13]. Such an energy band gap indicates that a more extended UV irradiation period is needed, as it leads to lower efficiency of the photodegradation process. According to Behrad and their colleague [14], TiO_2_’s photocatalytic activity was further affected by the fast backward reaction and recombination of electrons and holes generated by UV light, which is also known as photo-corrosion. The recombination of electrons and holes results in it losing the ability to undergo redox reactions by releasing energy as a photon or thermal energy [14,15]. To address such drawbacks, the use of heteropolyoxometalates (HPOMs) in this scenario could be beneficial.

HPOMs are the combination of inorganic polyoxometalates (large oxygen-enriched anionic building blocks (POMs)) and the functionalities of organic components [16]. POMs have broad applications in photocatalysis due to their structural diversity and outstanding physicochemical properties. The benefits of using POMs as photocatalysts are that they include many transition metals, such as Molybdenum (Mo), Tungsten (W), Vanadium (V), Niobium (Nb), and Tantalum (Ta), and that their surfaces contain a large number of potential active sites. Moreover, POMs can enhance photocatalytic performance by adjusting the band gap energy of POMs by changing the heteroatoms, which can include Phosphorus (P), Silicon (Si), Boron (B), Arsenic (As), or other elements. Furthermore, it can be functionalized to form HPOM by adding organic ligands or loading matrix materials such as carbon nanomaterials, which promote a synergistic interaction between other components [17].

POMs, metal ions, and organic ligands are the components commonly used to construct HPOM compounds. The metal ions are essential structural linkers connecting POMs and ligands [18]. HPOMs may also be readily synthesized or generated in situ by acidifying an aqueous solution under hydrothermal conditions [16]. This synthesis system under high pressure is advantageous when isolating crystals with dynamically stable structures [18]. The study of HPOMs has lower band-gap energy, which indicates that the HPOMs are easier to photoactivate than the semiconductor photocatalyst (such as TiO_2_), which can lead to higher photodegradation efficiency indirectly.

However, the direct contact between the photocatalyst nanoparticles and the dye solutions limits the reusability of photocatalyst nanoparticles, as they are scarcely separated from the treated water after wastewater treatment and were reportedly too small to be attracted by the magnet. The small amount of unremovable magnetic photocatalytic nanoparticles in the treated water has, in turn, created another water pollution problem due to the direct contact of the nanoparticles with the surroundings. In order to achieve a more sustainable future, this study is closely linked to Sustainable Development Goal 6 (SDG 6) on water and sanitation, which is a United Nations action plan that is expected to provide breakthrough technology for a better, safer, and more stable drinking water supply without generating secondary waste. Hence, newly developed magnetic-photocatalytic-responsive augmented microspheres (Fe_3_O_4_-TiO_2_ and Fe_3_O_4_-HPOMs), wherein the magnetic-photocatalytic particles are encapsulated within the polymer matrix, not only maintain high pollutant removal efficiency but also, most importantly, are able to separate the magnetic-photocatalytic microspheres from the treated water more wholly and conveniently.

The surface polyelectrolyte (poly(diallyldimethylammonium chloride) (PDDA)) functionalization of MNPs was employed to bind the negatively charged TiO_2_ or HPOM photocatalysts and encapsulated within the polymer matrix composed of polyethersulfone (PES) and polyvinylpyrrolidone additive (PVP) to form the proposed magnetic-photocatalytic microspheres with favorable properties. PES is considered one of the most important polymeric materials that are used in the synthesis of polymeric membranes using a phase inversion method for membrane water treatment. For example, in a work by Marjani et al. [19], graphene oxide-PES nanocomposite membranes were used to remove heavy metals such as zinc, cadmium, and copper ions. In a work by Mansor et al. [20], a tight ultrafiltration PES membrane was used to treat cheese-whey wastewater. Generally, PES is used as a base polymer because it provides unique properties, such as outstanding mechanical intensities, chemical resistance, thermal stability, and hydrolytic stability [21]. In addition, PVP acts as a viscosifier, which is essential for modifying the polymer properties [22]. As a result, it aids the creation of pores and can lend a hydrophilic character to the resulting polymer matrix [23]. The synthesized HPOM, f-MNP, and magnetic-photocatalytic composites were then subjected to elemental composition using energy dispersive X-ray spectroscopy (EDX) analyses as well as surface morphology and structure analyses using field-emission scanning electron microscopy (FESEM), which was followed by an assessment of the photocatalytic degradation efficiency of magnetic-photocatalytic-responsive microspheres. The synthesized microspheres’ reactive kinetic behavior (Langmuir–Hinshelwood model), reusability, and pH responsiveness were also elucidated.

## 2. Materials and Methods

### 2.1. Chemicals and Materials

Polyethersulfone (PES) polymer powder was purchased from BASF (Ludwigshafen, Germany), polyvinylpyrrolidone (PVP), N-methyl-2-pyrrolidone (NMP), silver acetate (AgC_2_H_3_O_2_), 1H-Benzotrailzole (Hbtz), 4-4′-Bipyridine (C_10_H_8_N_2_), sodium tungsten dihydrate (Na_2_WO_4_ ∙2H_2_O) were obtained from Merck (Darmstadt, Germany), iron oxide (Fe_3_O_4_) was supplied by NanoAmor (Houston, TX, USA), and poly(diallyldimethylammonium chloride) (PDDA), methyl orange, methyl blue and titanium dioxide (TiO_2_) by Sigma Aldrich (St. Louis, MO, USA). All the chemicals used were analytical grade.

### 2.2. Synthesis of HPOMs

First, 0.042 g of AgC_2_H_3_O_2_, 0.012 g of Hbtz, 0.015 g of C_10_H_8_N_2,_ and 0.121 g of Na_2_WO_4_∙2H_2_O were dissolved in 8 mL of distilled water in a beaker and stirred vigorously for 30 min. Then, the pH of the solution was adjusted to a range of 2 to 3 using HCl and H_2_SO_4_ before stirring again until a cloudy solution was formed. Afterward, the cloudy solution was poured into a Teflon-lined autoclave, and the heating temperature was operated at 160 °C for 3 days. Then, the solution was allowed to cool to room temperature before the washing process. The washing process was carried out using the centrifugation method, wherein the supernatant from the solution was poured out while the precipitates formed were washed with ethanol followed by distilled water. The washing process was repeated several times. Finally, the HPOM powder was obtained by drying it in an oven at 60 °C.

### 2.3. Fabrication of Magnetic-Responsive Microspheres

First, the PES polymer was added to the PVP and NMP solvent to obtain a PES-PVP polymer solution. The PES, NMP, and PVP were fixed at a weight ratio of 15/85/2.5. Then, different concentrations of MNPs at 0.1 wt.%, 0.2 wt.%, and 0.5 wt.% were added to the PES-PVP solution. The mixed solution was then channeled through a syringe needle to produce the PES-PVP-MNPs microspheres at the pumping speed of 0.1 mL/min using a Watson Marlow Peristaltic Pump.

### 2.4. Functionalization of MNPs with PDDA

The naked MNPs frequently aggregate in the solution due to the high surface energies, van der Waals, and magnetostatic interactions of MNPs that cause poor stability and dispersion [9]. Hence, in this work, the naked MNPs were first functionalized with poly(diallydimethylammonium chloride) (PPDA) to improve the MNPs’ stability and dispersion in water. The coating of PDDA on the MNPs’ surface (positive charge) could also help link the negatively charged TiO_2_ or HPOM via electrostatic attraction. A 0.0025 g/mL concentration of MNPs was suspended in distilled water and ultrasonicated for at least 30 min to obtain MNP suspension without large aggregates. Simultaneously, a 0.075 g/mL concentration of PDDA solution was ultrasonicated at 60 °C for 1 h to allow the PDDA to dissolve. The MNPs’ suspension and the PDDA solution’s pH were then adjusted to pH 8. The MNPs were then added to the PDDA solution in a dropwise manner. The binding of the PDDA to the surface of the MNPs was achieved through electrostatic attraction. The binding process was carried out in an orbital shaker at a speed of 150 rpm for 2 h. Before being dispersed into distilled water during the final step, the functionalized MNPs (f-MNPs) were first separated using a permanent magnet and prewashed several times. The successfully synthesized f-MNPs were then linked with TiO_2,_ or HPOM particles, as explained in Section 2.5.

### 2.5. Fabrication of Magnetic-Photocatalytic Microspheres

For the synthesis of the f-MNPs-TiO_2_ composite, 0.5 wt.% of f-MNPs were first dispersed in the same volume of distilled water as TiO_2_ particles (1.5 wt.%) to ensure the experiment was conducted under the same condition. The f-MNP suspension and the TiO_2_ suspension were adjusted to pH 8. The f-MNPs were then added to the TiO_2_ suspension in a dropwise manner. The binding process was carried out in an orbital shaker at a speed of 150 rpm for 24 h. Lastly, the f-MNPs-TiO_2_ composite was separated using a permanent magnet and prewashed several times before being dispersed into distilled water. Similarly, the steps used to synthesize the f-MNPs-HPOM composite were the same as the abovementioned steps. The formation of f-MNPs-TiO_2_ and f-MNPs-HPOM microspheres were the same as in Section 2.3.

### 2.6. Characterization of the Prepared Samples

The morphology of the prepared samples was examined using JEOL Field Emission Scanning Electron Microscopy (FESEM-JSM-6701F; Tokyo, Japan). All micrographs of the prepared samples were carried out at a magnification of 20,000×. A spectrum that displayed the peaks correlated with the elemental composition of the investigated samples was measured using an energy-dispersive spectroscopy system attached to a JEOL 6701F FESEM.

### 2.7. Photocatalytic Studies

**Figure 1:**
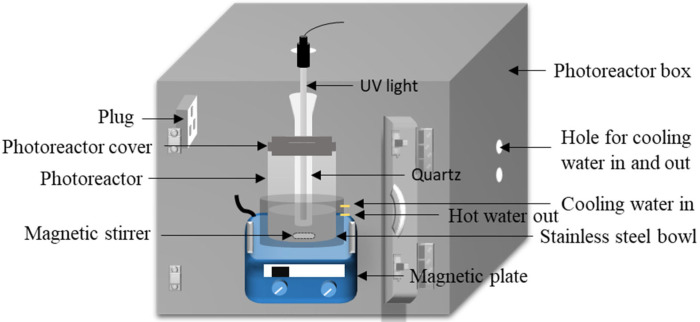
The photocatalytic reactor used in this study.

The dye solution was used as a model pollutant to evaluate the prepared sample’s photocatalytic efficiency for degrading the organic compound. The concentration (20 ppm) of the dye sample in 200 mL of dye solution was determined using the calibration curve of absorbance against the concentration prepared with a known concentration of UV-visible spectra. Absorbance measurement of MO dye was conducted using a UV-vis spectrophotometer at 465 nm. The photocatalytic degradation of dye aqueous solution with 0.5 g of microspheres was experimented with a photocatalytic reactor in darkness for 10 min and constantly stirred to obtain equilibrium between the solution and the catalyst. Subsequently, the solution was under UV irradiation for 90 min with continuous stirring (Figure 1). The samples were taken from the solution at 30 min intervals to determine the photocatalytic degradation efficiency of the pollutants. The efficiency of designed magnetic-photocatalytic microspheres for the degradation of MO was calculated as shown in Equation (1):

(1) $$Dye\;removal\;\left(\%\right)=\frac{C_{0}-C_{r}}{C_{0}}\times 100,$$

where $C_{0}$ and $C_{r}$ are the initial and residual concentrations of the dye solution (mg/L), respectively.

The effect of pH on f-MNPs-HPOM microspheres was investigated by varying the MO and MB dye solution to pH 3 and 9. Absorbance measurement of MB dye was conducted using a UV-vis spectrophotometer at 665 nm. The steps used to determine the photocatalytic degradation efficiency were similar to those mentioned above.

## 3. Results and Discussion

### 3.1. Magnetophoretic Separation Efficiency of Magnetic-Responsive Microspheres

**Figure 2:**
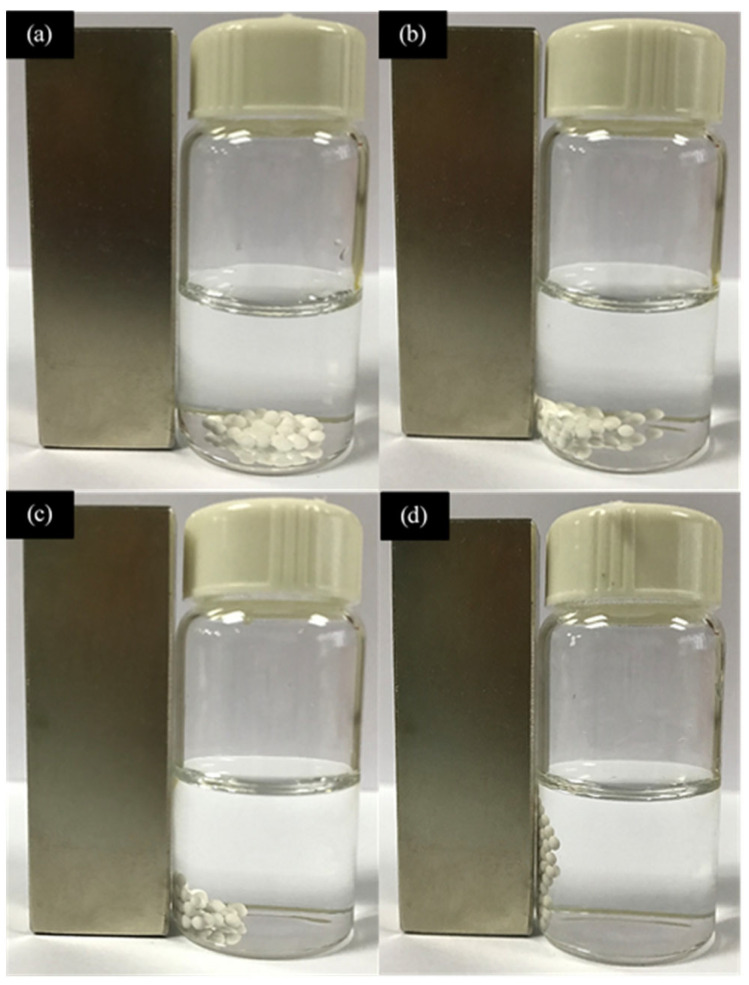
Magnetophoretic separation efficiency of (**a**) neat PESPVP microspheres, (**b**) PESPVP-MNPs0.1, (**c**) PESPVP-MNPs0.2, and (**d**) PESPVP-MNPs0.5 microspheres.

In investigating the effectiveness of the synthesized microspheres, various concentrations of Fe_3_O_4_ nanoparticles (MNPs) were added to the polymer solution to generate magnetic-responsive microspheres. As shown in Figure 2, all the microspheres except the neat PES-PVP microspheres (Figure 2a) responded to the external magnet, as they were attracted to the magnet. However, both PES-PVP-MNPs0.1 and PES-PVP-MNPs0.2 showed weak responses to the magnet, as they were only partially attracted by the external magnet (Figure 2b,c). This phenomenon might be due to insufficient MNPs to induce strong enough magnetic characteristics for magnetic attraction. As such, it can be deduced that the magnetic responsiveness of the microspheres increased with the increasing concentration of MNPs. Therefore, the PES-PVP-MNPs0.5 microspheres with the highest MNP concentration thoroughly responded to the external magnet, as all the microspheres were attracted toward the magnet (Figure 2d). In other words, PES-PVP-MNPs0.5 microspheres showed the highest recollect ability in the presence of a magnet.

### 3.2. Characterization of Synthesized Samples

**Figure 3:**
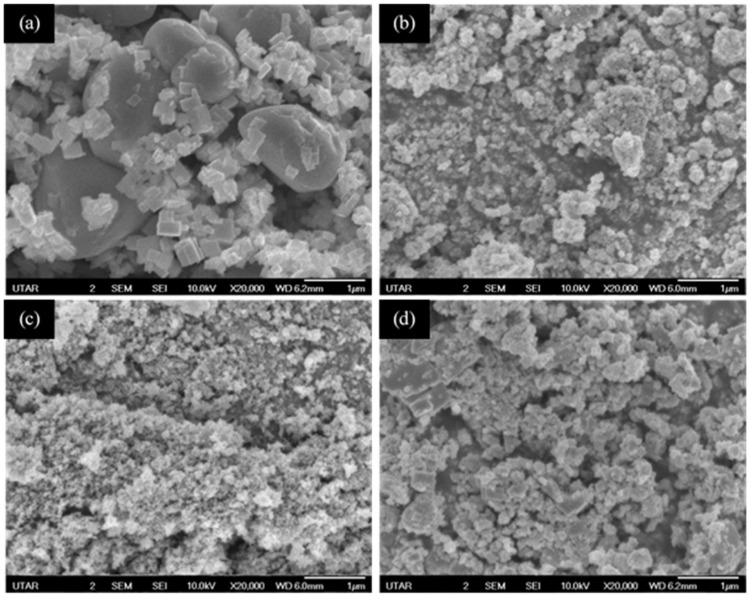
FESEM images of (**a**) HPOM, (**b**) f-MNPs, (**c**) f-MNPs-TiO_2_ composite, and (**d**) f-MNPs-HPOM composite at 20,000× magnification.

Before fabricating magnetic photocatalytic microspheres, it is crucial to evaluate the material characteristics of the synthesized HPOM, f-MNPs, f-MNPs-TiO_2_ composite, and the f-MNPs-HPOM composite. FESEM micrographs of the HPOM, f-MNPs, f-MNPs-TiO_2_ composite, and the f-MNPs-HPOM composite are illustrated in Figure 3. It can be observed that the shape of the synthesized HPOM particles (Figure 3a) consists of irregular spherical structures and crystal-like structures (with cubic, tetragonal, and orthorhombic shapes). Figure 3b shows the aggregated nanoparticles of the f-MNPs. This result does not reflect any colloidal instability of the f-MNPs, as the nanoparticles are prone to agglomeration by the strong surface tension of water during the drying process before FESEM analysis. Figure 3c,d illustrate a condensed layer of TiO_2_ and synthesized HPOM particles covering the f-MNPs in contrast to the f-MNPs (Figure 3b). Such an observation confirmed that a substantial amount of TiO_2_ and synthesized HPOM particles had successfully coated the surface of the f-MNPs.

These findings were further supported by EDX analysis, as shown in Table 1, which verified the existence of TiO_2_ and HPOM particles in the f-MNPs-TiO_2_ composite and the f-MNPs-HPOM composite, respectively. The elements present in HPOM included Carbon (C), Nitrogen (N), Oxygen (O), Silver (Ag), and Tungstate (W). These elements were originally from the reactants (AgC_2_H_3_O_2_, Hbtz, C_10_H_8_N_2_, and Na_2_WO_4_ ∙2H_2_O) of HPOM. The elements present in the f-MNPs were Carbon (C), Nitrogen (N), Oxygen (O), Chlorine (Cl), and Iron (Fe). After the incorporation of TiO_2_, a new element, Titanium (Ti), was detected in the f-MNPs-TiO_2_ composite, which had an atomic percent of 0.69. This confirmed the successful coating of TiO_2_ on the surface of the f-MNPs. Ag and W elements were detected in both the HPOM and the f-MNPs-HPOM composites, which had atomic percentages of 0.73, 1.09, and 0.02, 0.11, respectively. This conformation is crucial, as the W element plays a significant function as a transition metal atom in the formation of the HPOM structure. In contrast, for the Sodium (Na) element, the atomic percentage was found to be 0.00 in both the HPOM and the f-MNPs-HPOM composites. It is believed that the Na element was dissociated from the reactant sodium tungstate dihydrate and formed other compounds that would be discarded after the HPOM synthesis process. Thus, no detection of the Na element is available in both the HPOM and the f-MNPs-HPOM. Consequently, the presence of Ag and W and the absence of Na provided more evidence that the HPOM had been properly produced and coated on the surface of f-MNPs. Furthermore, the highest atomic percentage of the carbon element was found in the f-MNPs-TiO_2_ composite (63.13) and the f-MNPs-HPOM composite (64.22), which originated from aliphatic hydrocarbons found in the PDDA and HPOM particles. In conclusion, these results verified the successful synthesis of the HPOM, f-MNPs, f-MNPs-TiO_2_ composite and the f-MNPs-HPOM composite.

### 3.3. Photocatalytic Degradation Efficiency

**Figure 4:**
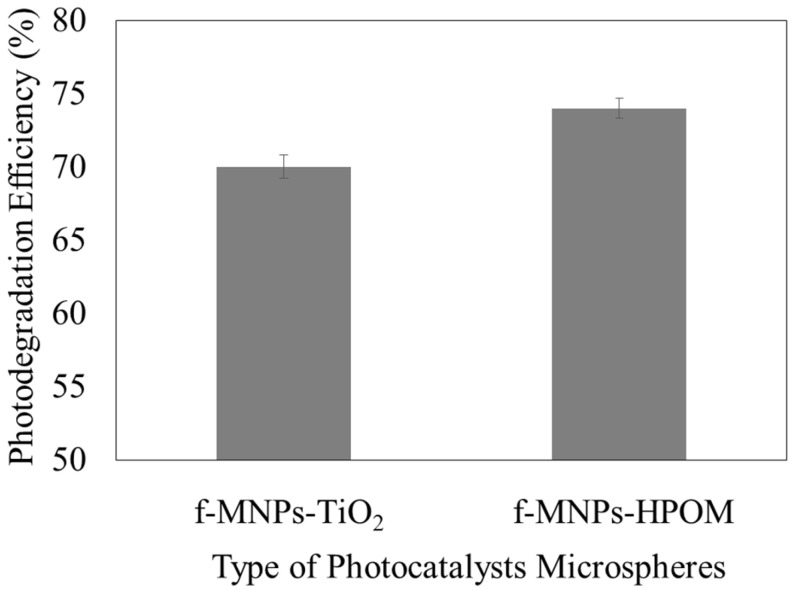
Photodegradation efficiency of f-MNPs-TiO_2_ and f-MNPs-HPOM microspheres.

**Figure 5:**
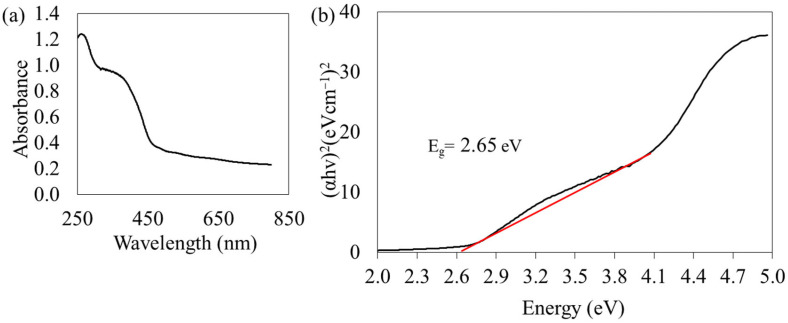
(**a**) UV-visible diffusion reflectance of the spectrum and (**b**) plots of (αhv)^2^ vs. energy of HPOM.

The photocatalytic activity of the magnetic-photocatalytic microspheres was evaluated using the photodegradation of methyl orange (MO) under UV-light irradiation for 90 min. Figure 4 shows the photodegradation efficiency of magnetic-photocatalytic microspheres. The photodegradation efficiency of the microspheres improved from f-MNPs-TiO_2_ to f-MNPs-HPOM microspheres under the same condition. For the f-MNPs-TiO_2_ and f-MNPs-HPOM microspheres, the presence of photocatalysts (TiO_2_ and HPOM) showed MO photocatalytic removal at 70 ± 0.8% and 74 ± 0.7%, respectively. The photocatalytic removal efficiency of f-MNPs-HPOM was higher than that of f-MNPs-TiO_2_. The band gap energies of both active catalytic sites are revisited to elucidate such differences in removal efficiency. The band gap energy of HPOM was found to be 2.65 eV, as displayed in Figure 5. Based on the literature, the band gap energy of TiO_2_ showed a higher value of 3.2 [11]. It is believed that the lower band-gap energy of HPOM enhanced the production of free hydroxyl radicals, which are essential to the degradation process in the UV region, as the wide band-gap energy of TiO_2_ is prone to having a fast recombination of electrons and holes generated by UV light, after which they lose their ability to undergo redox reactions to generate hydroxyl groups [11,14]. Consequently, higher MO removal efficiency was detected for f-MNPs-HPOM microspheres than for f-MNPs-TiO_2_ microspheres.

**Figure 6:**
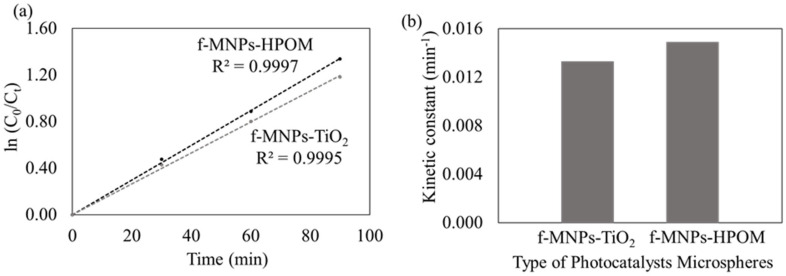
Corresponding plots for f-MNPs-TiO_2_ and f-MNPs-HPOM microspheres (**a**) The pseudo-first-order reaction and (**b**) kinetic constants for photocatalytic degradation for MO.

The photocatalytic degradation kinetics of MO were investigated to gain insight into the reactive kinetic behavior of MO degradation in the photocatalyst microspheres. The kinetic curves of MO degradation using either photocatalyst microsphere are well-fitted to the Langmuir–Hinshelwood pseudo-first-order kinetic model (R^2^ > 0.99), wherein (*C*_0_/*C_t_*) was directly proportional to the contact time, as displayed in Figure 6a. Additionally, the kinetic constant is higher for the f-MNPs-HPOM microspheres than for the f-MNPs-TiO_2_ microspheres, at 0.0149 min^−1^ and 0.0133 min^−1^, respectively (Figure 6b). Hence, it can be concluded that the degradation of MO with f-MNPs-HPOM is not only faster (higher rate constant) but better (higher removal efficiency) than that with f-MNPs-TiO_2_ (Figure 4).

**Figure 7:**
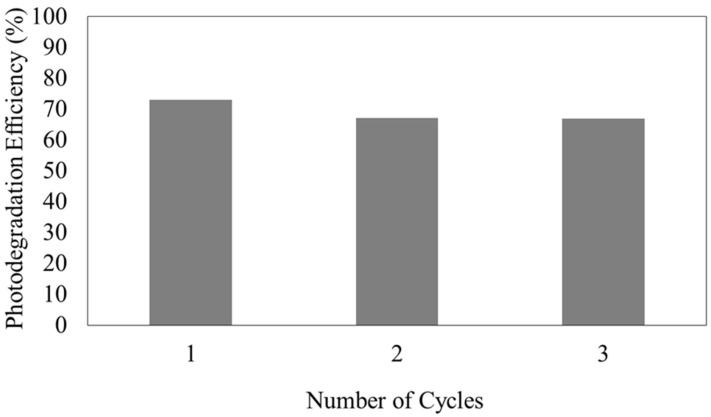
Photocatalytic removal efficiency in reusability cycles of f-MNPs-HPOM microspheres.

The stability of a photocatalyst is of major concern; thus, the reusability of HPOM microspheres for MO photodegradation with UV light was evaluated for three cycles. After 90 min of irradiation, the microspheres were separated from the reaction mixture using a magnet, washed with distilled water, and reused for repeated MO photodegradation tests. As shown in Figure 7, only a nominal and gradual decrease in the photocatalytic performance could be observed, and nearly 70% photodegradation efficiency was retained throughout the three cycles. The slight reduction in photodegradation efficiency during the second and third cycles might be due to the reduced active site on the surface of photocatalyst particles. The microsphere surface could be saturated with the deposition of MO, which caused fewer photons to be available to reach the microsphere surface, and ultimately caused the decrease in the formation of free hydroxyl radicals [24].

In addition, the residual MO adsorbed on the photocatalyst surface might not be completely removed during the regeneration process. Hence, a slightly smaller surface area of HPOM was exposed to UV light, causing a decrease in the photocatalytic activity, and ultimately reducing the MO’s removal efficiency. Nevertheless, such a decrease in photocatalytic activity was only nominal; thus, it can be confirmed that f-MNPs-HPOM exhibited excellent reusability for photodegradation of MO under the reaction condition for at least three cycles. Kaur et al. (2021) also encountered a similar circumstance, wherein the reported MO degradation efficiency reduced slightly during the second and third cycles.

In addition, the small reduction in photocatalytic degradation efficiency of f-MNPs-HPOM microspheres was compared with the values reported in the literature. For example, in a work by Kaur et al. [24], H_2_O_2_ regeneration techniques were used to treat the photocatalysts, and the photodegradation efficiency of cypermethrin after three cycles was reduced by nearly 4%. In a work by Karimzadeh et al. [25], after three cycles of photocatalysts’ regeneration, the efficiency was reduced by 6%. Based on a comparison with the literature, it can be said that the f-MNPs-HPOM microspheres prepared in this experiment had better reusability and regeneration performance than that found in the literature, which was approximately 3%.

### 3.4. pH Responsiveness Evaluation of the Prepared f-MNPs-HPOM Microspheres

**Figure 8:**
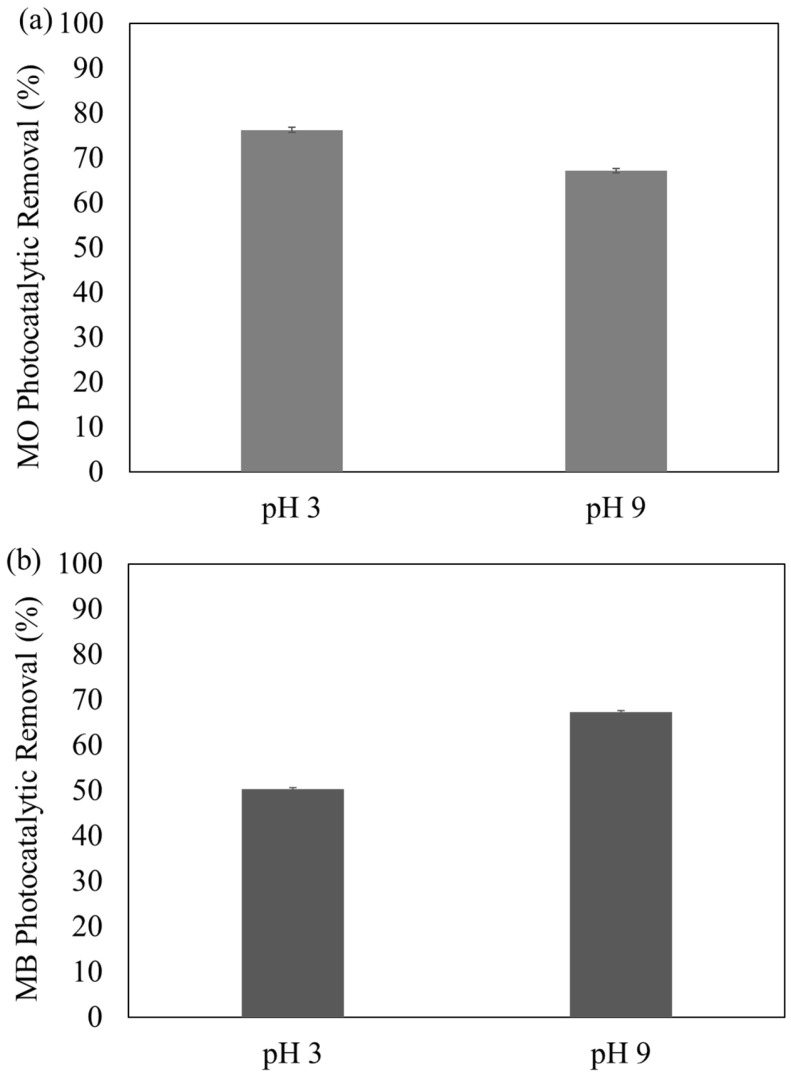
The pH-responsiveness of f-MNPs-HPOM microspheres in photodegradation of (**a**) MO and (**b**) MB.

As indicated in Figure 8a, f-MNPs-HPOM microspheres showed better adsorption at pH 3 (76 ± 0.5%) than at pH 9 (67 ± 0.5%). Such a phenomenon could be due to more proton (H^+^) on the surface of the f-MNPs-HPOM microspheres at pH 3 [26]. Subsequently, the MO, an anionic dye molecule (negative charge), attracted the positively charged f-MNPs-HPOM microspheres and improved the interaction between the adsorbate and adsorbent, which ultimately led to high MO photocatalytic degradation efficiency at a low pH. On the contrary, in the basic solution, due to electrostatic repulsion on a similar charge, the rate of adsorption of MO on f-MNPs-HPOM microspheres decreased, thereby lowering the rate of photodegradation. Similar results were also obtained by Hanafi et al. [27], who found that the photocatalysts used accelerated the decolorization of the MO solution at a low pH.

The same concept could also be applied to the degradation of MB. MB is a cationic dye molecule (positive charge) that is attracted to the negatively charged f-MNPs-HPOM microsphere at pH 9. At pH 9, the surface of the f-MNPs-HPOM microspheres is susceptible to being negatively charged due to a reaction with hydroxyl (OH^-^) ions [28]. Consequently, when the interaction between MB (positive charge) and f-MNPs-HPOM (negative charge) was promoted, high photocatalytic decolorization efficiency of f-MNPs-HPOM microspheres at pH 9, 67 ± 0.4% could be detected, as shown in Figure 8b. Conversely, a similar charge obtained using an acidic solution caused electrostatic repulsion, thereby lowering the rate of adsorption of MB on the f-MNPs-HPOM microspheres, and ultimately resulting in the decrease in photodegradation efficiency (50 ± 0.2%). The same outcomes were also obtained by Chaudhari et al. [29], who found that the photocatalysts used had hastened the MB color degradation in high pH solutions. These results align with the results of this study and its conclusion that the synthesized f-MNPs-HPOM microspheres were indeed pH-responsive.

## 4. Conclusions

A new photocatalyst, HPOM, which has the potential to perform better than TiO_2_ in the photocatalytic processes, was synthesized. The successfully development of HPOM was proven using FESEM and EDX analyses. The calculated band-gap energy of HPOM was 2.65 eV. The f-MNPs-HPOM microsphere showed a higher photocatalytic degradation performance of 74% more than the f-MNPs-TiO_2_ microsphere. The results highlighted how the photocatalysts with lower band-gap energy raised the photodegradation efficiency of MO. The Langmuir–Hinshelwood pseudo-first-order kinetic model well-fitted the kinetic behavior of MO degradation in f-MNPs-TiO_2_ and f-MNPs-HPOM microspheres. The f-MNPs-HPOM microsphere presented the highest kinetic constant at 0.0149 min^−1^. As reported, f-MNPs-HPOM microspheres exhibited excellent reusability for the photodegradation of MO, as there was no noticeable decrease in the photocatalytic performance observed and they were retained at nearly 70% throughout the three cycles. Finally, MO was more suitable for photodegrading by the f-MNPs-HPOM microsphere in the acidic pH 3 condition, as it showed a photodegradation efficiency of 76%. In the case of MB, the f-MNPs-HPOM microsphere showed better photocatalytic removal of 67% at pH 9. These results further strengthen the claim that the synthesized f-MNPs-HPOM microspheres are indeed pH-responsive. In fact, the newly developed photocatalyst with magnetic properties was incorporated into a PES polymer matrix, which is a well-known polymer used in the fabrication of polymeric membranes for membrane applications. Furthermore, most of the polymeric membranes in the literature have problems with membrane fouling. Therefore, it is possible to apply the newly developed photocatalyst incorporated into the membrane to control membrane fouling, which can be studied and investigated in the future. Additionally, the f-MNPs-HPOM microspheres developed in this work were able to degrade organic pollutants and successfully demonstrated responsivity toward the tri-functionalities of pH, magnetic, and photocatalytic. Consequently, this work demonstrates that the developed magnetic photocatalyst has the potential to be applied in the synthesis of a photocatalytic polymeric membrane. As such, it is strongly believed that this research can help with the development of highly reusable or sustainable photocatalytic polymeric membranes in the future.

## Figures and Tables

**Table 1:** The atomic percentage of the elements present in the HPOM, f-MNPs, f-MNPs-TiO_2_ composite, and the f-MNPs-HPOM composite.

Element	Atomic (%)			
	HPOM	f-MNPs	f-MNPs-TiO_2_ Composite	f-MNPs-HPOM Composite
C	61.94	40.35	63.13	64.22
N	10.24	2.66	0.00	12.18
O	25.99	49.01	33.95	22.44
Cl	0.00	0.08	0.00	0.02
Ag	0.73	0.00	0.00	0.02
W	1.09	0.00	0.00	0.11
Fe	0.00	7.91	2.23	1.01
Ti	0.00	0.00	0.69	0.00

## Data Availability

Not applicable.
